# TADMaster: a comprehensive web-based tool for the analysis of topologically associated domains

**DOI:** 10.1186/s12859-022-05020-2

**Published:** 2022-11-04

**Authors:** Sean Higgins, Victor Akpokiro, Allen Westcott, Oluwatosin Oluwadare

**Affiliations:** Department of Computer Science, University of Colorado, Colorado Springs, CO USA

**Keywords:** Hi-C, Chromosome conformation capture, Topologically associated domains, TADs

## Abstract

**Background:**

Chromosome conformation capture and its derivatives have provided substantial genetic data for understanding how chromatin self-organizes. These techniques have identified regions of high intrasequence interactions called topologically associated domains (TADs). TADs are structural and functional units that shape chromosomes and influence genomic expression. Many of these domains differ across cell development and can be impacted by diseases. Thus, analysis of the identified domains can provide insight into genome regulation. Hence, there are many approaches to identifying such domains across many cell lines. Despite the availability of multiple tools for TAD detection, TAD callers' speed, flexibility, result inconsistency, and reproducibility remain challenges in this research area.

**Results:**

In this work, we developed a computational webserver called TADMaster that provides an analysis suite to directly evaluate the concordance level and robustness of two or more TAD data on any given genome region. The suite provides multiple visual and quantitative metrics to compare the identified domains' number, size, and various comparisons of shared domains, domain boundaries, and domain overlap.

**Conclusions:**

TADMaster is an efficient and easy-to-use web application that provides a set of consensus and unique TADs to inform the choice of TADs. It can be accessed at http://tadmaster.io and is also available as a containerized application that can be deployed and run locally on any platform or operating system.

**Supplementary Information:**

The online version contains supplementary material available at 10.1186/s12859-022-05020-2.

## Background

Chromosomes are known to self-organize into nonrandom three-dimensional (3D) structures in distinctive territories in a nucleus [1, 2]. Within these territories, chromosomes form other structures, such as topologically associated domains (TADs) [3, 4], smaller sub-TADs [5, 6], and chromatin loops [7]. These structures have been verified by techniques, such as chromosome conformation capture [3] and subsequent high-throughput technologies, such as Hi-C [8]. TADs have been identified as a key structural and regulatory unit of the genome [4, 9, 10]. The locations of TADs are largely invariant between cell types and species [4, 9–12], which alludes to their evolutionary importance. However, recent research has revealed that TADs and other genomic structures can be altered [13–16]. For example, TAD boundaries have been altered in Drosophila through heat shock, which resulted in TAD merging [13]. Furthermore, TAD boundary disruptions have been associated with various human diseases and cancer [17–19]. Due to the importance of TADs, numerous tools (or TAD callers) have been developed for identifying these genomic structures. TAD callers' approaches to finding TADs can loosely be classified as linear scoring, clustering, or statistical modeling [20, 21]. The variety of approaches can lead to differing speeds and result consistency. Additionally, it is common for callers to be designed and/or optimized for a particular genomic dataset, which can impact the method's flexibility when applied broadly. Thus, it is challenging to determine which set of TADs is closest to the ground truth.

**Fig. 1:**
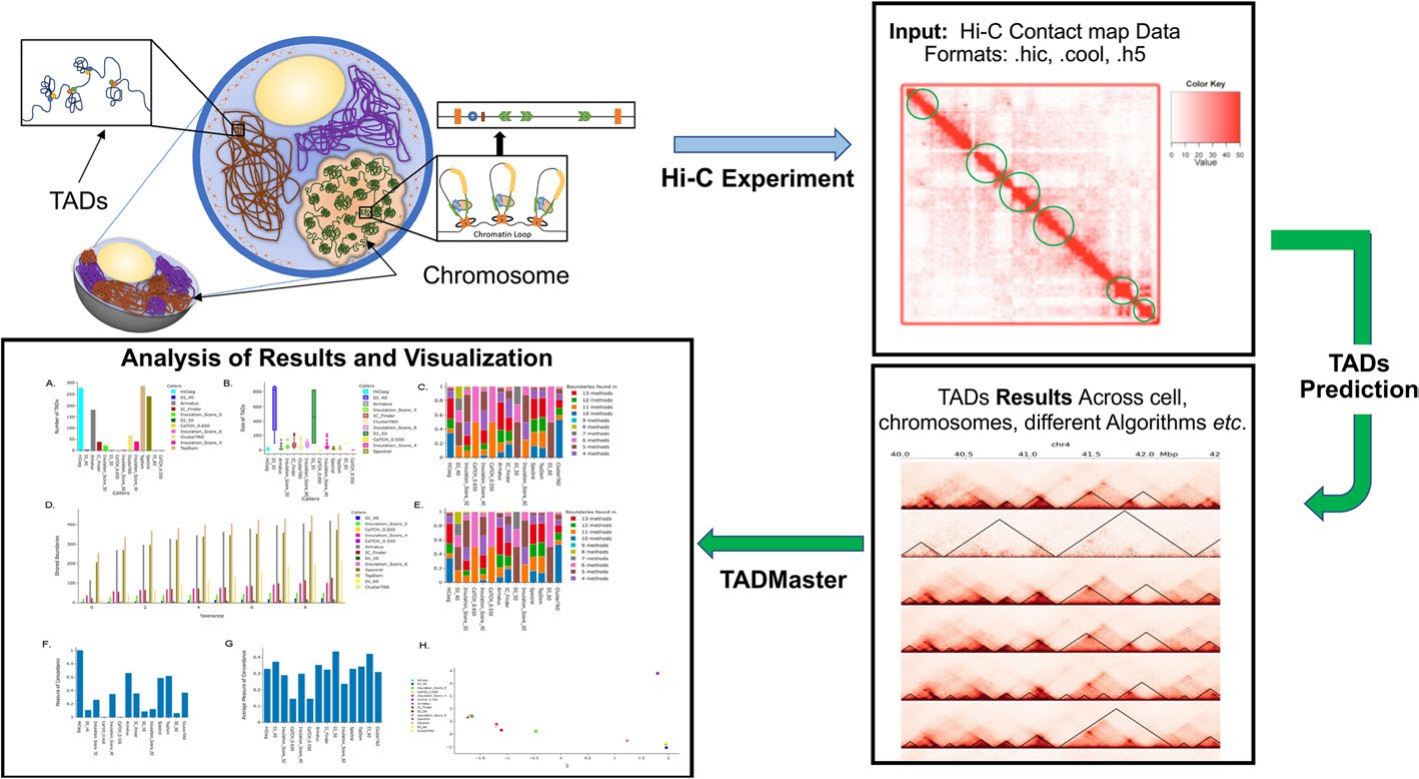
Graphical Abstract of TADMaster’s Motivation. A graphical abstract illustrating the motivation for TADMaster, beginning from the Hi-C experiment to the TADMaster output

To provide easy accessibility and comparative analysis of different TAD results, we introduce a webserver called TADMaster, which allows users to upload their own TAD data, for instance, across different cells, chromosomes, or algorithms, for comparative analysis (*see Fig. **1*). TADMaster provides an analysis suite that evaluates the concordance level and robustness of two or more TAD datasets. The latter is accomplished by providing numerous points of comparison between the provided data. TADMaster's analysis includes a quantitative comparison of the size/number of identified regions, the boundaries of the identified regions, the totality of the domains, and the amount of domain overlap for comparison between different TAD data. TADMaster shows, via graphs, the correlations and similarities between different TAD results through a clustered map. Previous work has evaluated TADs on similar metrics; however, they did not provide a generalized analysis tool that can be applied to an arbitrary genomic dataset [20]. Furthermore, the tools that do allow for an arbitrary dataset only provide a limited subset of the analysis provided by TADMaster [22, 23]. For example, TADMaster is the only tool available that performs an on-demand measure of concordance comparison of TAD datasets.

**Fig. 2:**
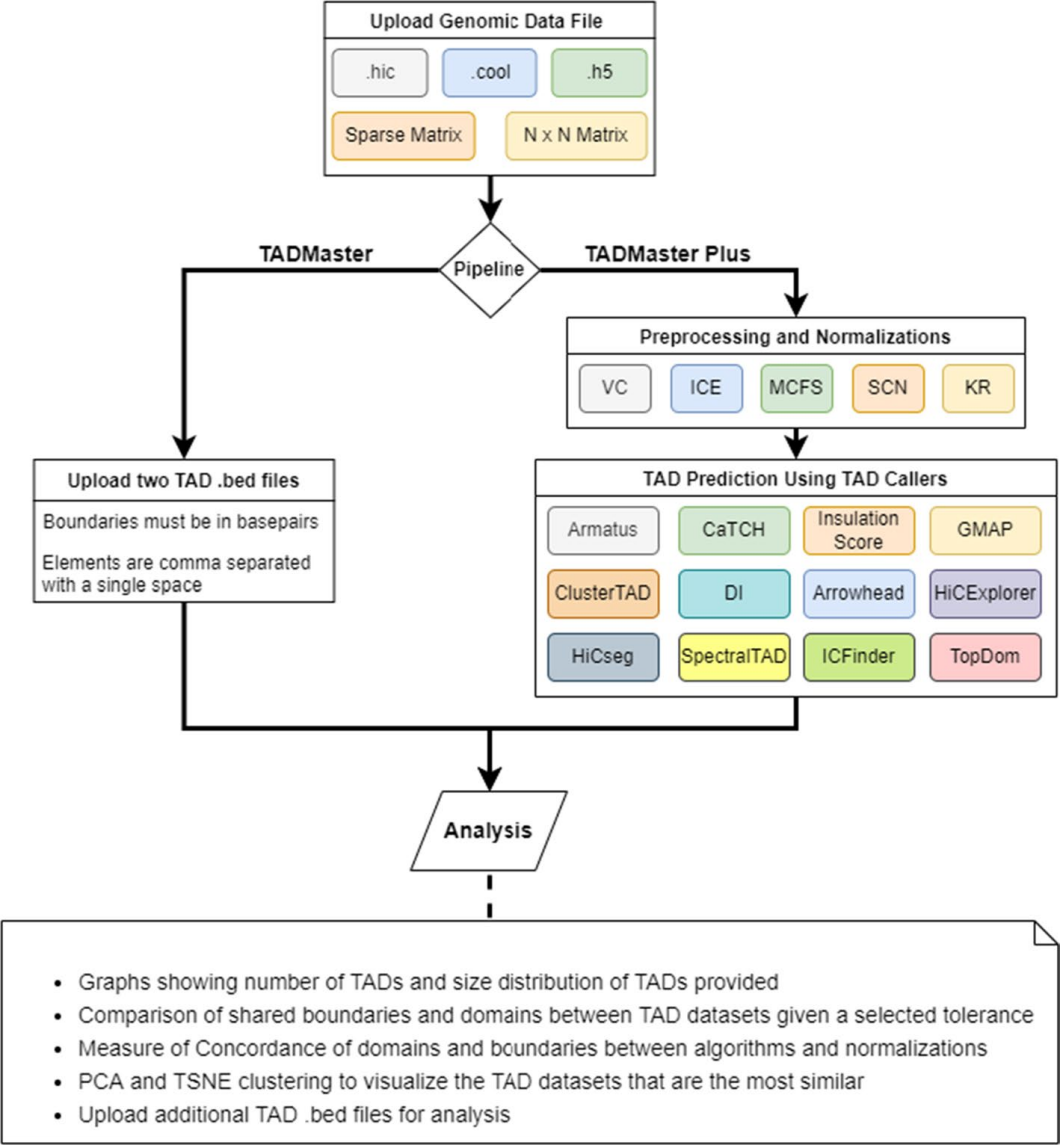
TADMaster and TADMaster Plus Pipeline. TADMaster (left) and TADMaster Plus (right) pipelines with descriptions of the accepted input file types, possible operations, and analysis results

Furthermore, TADMaster provides a "Plus" service that performs up to five normalizations and includes twelve state-of-the-art TAD callers on genetic data to provide a starting point for researchers, experts and nonexperts for a comparative analysis of these TAD callers' results on input cell data. The TADMaster and TADMaster plus pipelines are depicted in Fig. 2. We provide the details about the normalization and TAD callers included in the TADMaster Plus pipeline in the Methods section of the Additional file 1: Supplementary document.

### Implementation

TADMaster is a webserver (http://tadmaster.io) with a user-friendly graphical interface. A link to a comprehensive user guide, tutorial, and example datasets are provided on the website. TADMaster’s only input requirement is a TAD dataset that will be used for the comparative analysis. If the provided dataset is in a compressed form, such as cool or h5, TADMaster will utilize the metadata to extract the specific chromosome that is specified by the user.

TADMaster was designed based on a client–server architecture. On the client side, the user interface was built with HTML 5 for content, CSS for layout and style, JavaScript for website activity, and AJAX for increased usability. For the user's job submission management, we utilized MySQL as the database engine. To allow for clean and speedy design and development, the server side was written in Python using the Django framework. The backend task analysis, data storage, and analysis are handled by the server on job submission. For the backend, Python is used to implement the features and general criteria. Other elements, such as data normalization and data file format conversion, were handled by the server. The plot displays are handled by the browser-based client. TADMaster assists scientists and researchers with easy comparison, analysis, and visualization by providing much on-the-go information while they hover over the analysis plots.

### Visual and quantitative

**Fig. 3:**
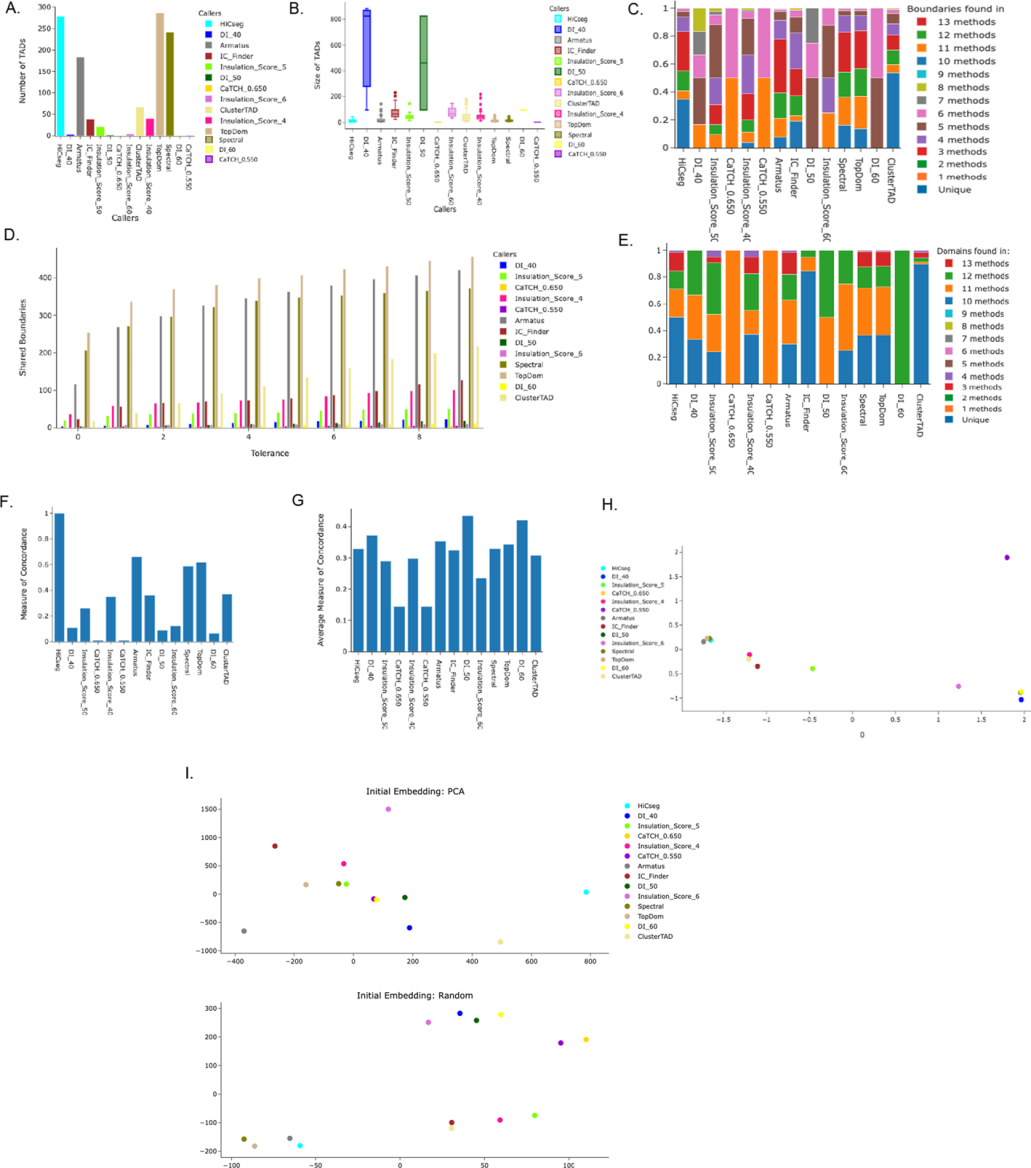
TADMaster Analysis Suite. A sample of the analysis results provided by TADMaster on chromosome 10 from human embryonic stem cell (hESC) at 40 KB resolution from multiple TAD datasets. TAD identification method comparison based on **A** the number of TADs identified, **B** the size of TADs identified, **C** the number of shared boundaries with a tolerance of one, **D** a one-versus-all comparison of the number of shared boundaries, **E** the number of shared domains with a tolerance of one, **F** a one-verse-all MoC comparison of domain overlap, **G** an all-verse-all MoC comparison of average domain overlap, **H** PCA clustering to group the TAD dataset and **I** TNSE clustering to group the TAD dataset based on dataset similarity

TADMaster provides a comparison of the number and size of TADs found in each method (Fig. 3A and 3B). The former is displayed in a bar diagram. The latter is displayed in a standard box and whisker plot to provide better context of the range and consistency of TAD sizes each method identifies. TAD size information is reported in terms of genomic bins whose size is determined by the supplied resolution.

### Boundary and domain

The analysis includes a comparison of the number of shared boundaries given a certain margin of error or tolerance (Fig. 3C and 3D). The tolerance is determined by the resolution of the dataset provided and is adjustable by the user. TADMaster uses tolerance as a metric for the margin of error for comparison between TADs. Tolerance is defined by the resolution provided by the user when the job is created. For example, when comparing the number of shared TAD boundaries, at a tolerance of zero, boundaries have to be identical to be counted as shared; at a tolerance of one, boundaries must be within one genomic bin (plus or minus 1 $\times$ the resolution) to be counted as shared. Additionally, comparing the number of TAD domains is similar, but both the rising and falling boundaries of the TAD domain must fall within the selected tolerance of the domain that it is being compared with. A range of tolerances is provided for each visualization. It is important to consider the average size of TADs when analyzing the results of the number of shared boundaries and domains. The number of shared boundaries is presented as a raw count in a one-verse-all graph and an all-verse-all percentage-based stacked bar graph (Fig. 3C). Similarly, TADMaster includes an all-verse-all comparison of the number of shared domains, where both the start and end of the TADs being compared must be equivalent given a particular tolerance (Fig. 3E).

### Concordance analysis

**Fig. 4:**
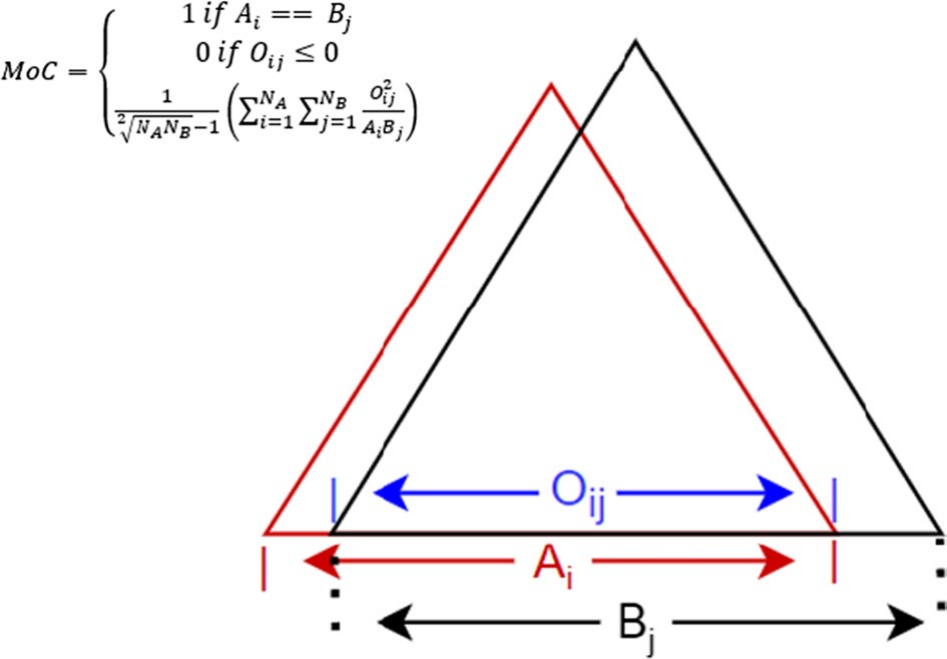
Measure of concordance (MoC) Calculation. Graphical illustration of the calculation for the measure of concordance with the relevant formula, where ${{\varvec{N}}}_{{\varvec{A}}}$ and ${{\varvec{N}}}_{{\varvec{B}}}$ are the respective number of TADs from two datasets

Pfitzner, D. et al., 2009 [24] performed a comprehensive study of different measures and metrics for comparing sets of partition, including the Jaccard index, overlap coefficient, VI, and Mountford, to determine the goodness of clustering and the similarity of clustering by clarifying the degree to which different measures confuse the two. Pfitzner, D. et al. found that the measure of concordance (MoC) produced the best results and satisfied the desired behavior of a similarity measure, which represented the difference between partitions under various testing conditions. In addition, a recent comprehensive study of TAD algorithms by Zufferey et al., 2018 [20] used the MoC measure to perform an analysis of the differences between the TAD overlaps performed in their work. Because of MoC strength and relevance in the chromatin genomics area, we employed the MoC metric to quantify concordance between TADs in this work. The measure of concordance (MoC) is also evaluated for each method by determining the domain overlap of the TADs for each method. MoC is calculated by taking each method and comparing it iteratively to all other methods. The amount of overlap is calculated by squaring the overlapping region and dividing it by the product of the size of the original TADs (Fig. 4). The result of this evaluation is first presented in a one-verse-all plot, where the value is the percentage of overlap with the selected method. The measures of concordance for each individual method with regard to all other methods are also averaged and presented in an all-vs.-all manner. The latter visualizes each method’s relative agreement with all other methods in terms of domain overlap.

The MoC is evaluated for each TAD dataset by determining the domain overlap of the TADs for each method. The amount of overlap is calculated by squaring the overlapping region and dividing it by the product of the size of the original TADs (Fig. 4). The result is first presented in a one-verse-all plot (Fig. 3F), where the MoC of each dataset is compared to the one piece of data selected. Additionally, an all-verse-all plot is provided, which shows the average MoC of each dataset compared to all other provided datasets (Fig. 3G).

### Clustering

TADMaster also uses principal component analysis (PCA) (Fig. 3H) and t-distributed stochastic neighbor embedding (TSNE) (Fig. 3I) to perform clustering based on the provided TAD data. Both approaches group methods based on their respective domain similarities.

## Results

A selected subset of a typical analysis provided by TADMaster is depicted in Fig. 3, where seven TAD datasets from human embryonic stem cell (hESC) chromosome 10 at 40 KB resolution [4] are compared. The number of TADs graph (Fig. 3A) shows the comparison of the fourteen TAD datasets from different TAD Callers. The description of the TAD Callers and implementation details are provided in the supplementary document (Additional file 1: Methods). The size of the TADs identified (Fig. 3B) was observed to be inversely related to the number of TADs. The number of shared boundaries (Fig. 3C) demonstrates that all datasets found a small number of boundaries that were also identified by all other methods, 3 methods depicted in red. From the plots, four datasets have the highest number of TADs identified: 286 (TopDom [25]), 276 (HiCseg [26]), 241 (Spectral [27]), and 183 (Armatus [28]) (Fig. 3A). Figure 3B shows that, however, these four datasets have a consistent TAD size. Of the four, Armatus reported the fewest unique boundaries identified.

(Fig. 3C) and the fewest unique domains among the four identified TAD datasets (Fig. 3E). However, as shown on the plot in the red color legend ( annotating 3 methods), each of this four method’s boundaries and domains have a high correlation with three other methods (Fig. 3C and 3E). These can be construed to mean that, while the number of TADs found is lower than the others, it reports less distinct TADs detected and higher overlaps with other TAD datasets. The comparison of the number of shared boundaries between the various datasets on a one versus all criteria at different tolerance degrees, in this case HiCSeg versus other methods, shows that the boundaries shared with TopDom are consistently high across different tolerance degrees (Fig. 3D). However, the MoC of HiCseg versus all other methods shows that these four TAD datasets are similar (Fig. 3F), and the average MoC of each dataset also mirrors the previous results, with these datasets having a consistent amount of concordance on average (Fig. 3G). Furthermore, these methods form the only tight cluster in the TSNE and PCA comparison (Fig. 3H and 3I). The results presented here can be seen live at this link: http://biomlearn.uccs.edu/TADMaster/visualize_example/415/. Ultimately, from this result, one can deduce a consensus and unique pattern between the TAD dataset results rather than considering them in isolation.

Finally, we performed a time-based performance analysis of TADMaster, which is available in Supplementary Table S1 (Additional file 2) and Table S2 (Additional file 3). The former denotes the expected load times of the visualization tool based on the size of the unpacked square matrix from the TAD dataset. The results show that load time is relatively constant for most chromosomes but is doubled on large chromosomes (e.g., chromosome 1). Table S2 (Additional file 3) provides runtime data for TADMaster Plus based on the method selected on chromosome 8 (with size = 26,292 KB) and chromosome 19 (with size = 5,000 KB) on high-resolution Hi-C data. The time performance of the methods is directly correlated to the time complexity of each TAD identifying method to show how long each algorithm takes on each job. The TADMaster web server runs on a HP G7-DL980, Intel(R) Xeon(R) CPU E7- 4870 @ 2.40 GHz server with 120 Cores,1 Terabyte (TB) of RAM and 40 TB of storage space. In addition, for users to take advantage of their own hardware resource capabilities to improve computational performance, TADMaster can be run locally using the local containerized version we provide to take advantage of any advanced hardware resources. A step-by-step instruction for using this local containerized version is provided on TADMaster’s GitHub repository.

## Discussion

When evaluating the TADMaster analysis, it is important to compare the various results provided. As indicated in the results, four TAD datasets demonstrated the highest level of agreement across most metrics. This does not signify correctness or superiority in our perspective; however, these TAD datasets are likely a good starting point for comparative analysis of this genomic dataset. In addition, the TADMaster analysis provides insight into possible reasons why the other datasets are in less agreement. We can consider a measurement of agreement established by Zufferey et al. The DI [4] TAD dataset (*e.g.,* DI_40) was shown to find fewer large TADs but recorded the highest average MoC value of 0.37. The latter demonstrates that this dataset identifies the exact genomic spans but identifies larger genomic structures. Conversely, the HiCseg dataset was shown to find many small TADs with an average MoC value of 0.33. TADMaster provides an avenue for future study of the agreement between these TAD datasets. This study is significant because TADMaster reports will reveal key biological insights. It is impossible to judge the accuracy of TAD callers' and/or TAD datasets because it is difficult to achieve a consensual agreement for several methods on the same data. Furthermore, there is no experimental dataset available for benchmarking and labeling, and the validation of existing results relies on identifying functional regulatory elements found in TAD boundaries in the human genome. Thus, TADMaster's analysis focuses on computational analysis of TAD results. It provides multiple points of comparison for determining the similarities between TAD datasets but does not provide a metric or suggest the most accurate TAD data. However, by comparing all the provided results, researchers can quickly identify self-similar datasets as a reference point for further biological research to make appropriate conclusions.

## Conclusions

Topologically associated domains (TADs) play a key role in genomic expression. Furthermore, the identification of such regions from Hi-C data plays a key role in the understanding of genomic diseases. There have been dozens of publications for determining the location of TADs; however, it is common to have conflicting results between TAD identifying methods. Further the abundance of genomic data and algorithms for determining loci make it imperative to illustrate a consensus between methods. Thus, we analyze information from several data sources to understand consensus and unique TAD regions, thereby reducing some of the drawbacks of relying on single sources for high-quality results. TADMaster aims to achieve this goal by providing an easy-to-use online web server platform that works across multiple operating systems and browsers that supports a side-by-side comparison and visualization of the multiple TAD results. TADMaster displays the results about the degree of consensus between different TAD datasets uploaded to identify consensus, uniqueness, and differences in results among cells, chromosomes or TAD results from different TAD callers or methods, thus overcoming some limitations in overly relying on a computationally generated result from single sources and thereby improving the reliability of the results. Additionally, we create a containerized version of the TADMaster webserver that can be run on any platform using Docker. This containerized version has the functionality of the server so that individuals can process jobs on their local systems. With this local version, there are no size limitations, as they will be run locally on the user’s machine. Other benefits of this local version include users having the ability to use their available resources to run jobs immediately without joining the job queue, can easily extend or modify the code to meet their needs, and, most importantly, users will save a considerable amount of time installing dependencies and using the server on the go because of the Docker image provided.

### Availability and requirements

Project name: TADMaster

 Project home page: http://tadmaster.io

 Operating system(s): Platform Independent

 Programming language: HTML, CSS, JavaScript, AJAX, MySQL, Python, and R. Other requirements: Conda, Docker. License: GNU GPL v3

Any restrictions to use by non-academics: None.

## Supplementary Information

**Additional file 1: **Supplementary note providing a description of each TAD caller, the parameters used in this study, the normalization algorithms, and the running time estimates for the results.

**Additional file 2: Table S1. **Initial time to load visualize analysis results from a sample of chromosomes with between 12 to 14 TADs datasets displayed.

**Additional file 3: Table S2.**Comparison of time to run the Normalization and TADCaller algorithms between chromosome 8 (with size = 26,292 KB) and chromosome 19 (with size = 5000 KB). The chromosomes were provided in square matrix formats, and both chromosomes were run with all normalization methods selected.

## Data Availability

TADMaster is a free web-based application open to all users with no login required at http://tadmaster.io. All our source code, data and documentation tutorials are available at https://github.com/OluwadareLab/TADMaster and are made available as a containerized application that can be run on any platform.

## Abbreviations

3C: Chromosome conformation capture
IF: Interaction frequency
MoC: Measure of concordance
PCA: Principal component analysis
TSNE: T-distributed stochastic neighbor embedding
