# Supplementary material for: TADMaster: a comprehensive web-based tool for the analysis of topologically associated domains
Source: BMC Bioinformatics. 2022 Nov 4;23:463. doi: 10.1186/s12859-022-05020-2 (PMC9636664; doi:10.1186/s12859-022-05020-2)
Supplement: Supplementary file 1 — Additional file 1: Supplementary note providing a description of each TAD caller, the parameters used in this study, the normalization algorithms, and the running time estimates for the results. [file 12859_2022_5020_MOESM1_ESM.pdf]

Supplementary Document

TADMaster: A Comprehensive Web-based Tool for the Analysis of Topologically Associated Domains

Sean Higgins[1], Victor Akpokiro[1], Allen Westcott[1], and Oluwatosin Oluwadare[1,*]

[1] Department of Computer Science, University of Colorado at Colorado Springs, Colorado Springs, CO, 80918, USA

*To whom correspondence should be addressed.

Contact: ooluwada@uccs.edu

Introduction

TADMaster is a web-based application that provides visualization/analysis of topologically associated domains (TADs) and provides comprehensive comparison of the methods that identify them. TADMaster has two pipelines. The “TADMaster” pipeline provides an analysis suite for comparing two TADs datasets. The “TADMaster Plus” pipeline which takes a user-provided contact matrix and utilizes state-of-the-art normalization and TAD identification methods in order to generate TADs datasets. This pipeline also includes the TADMaster TAD analysis on the resulting datasets. This document will provide further details of the implementation, parameter settings for algorithms, analysis, and estimated run times.

Methods

Hi-C Data Formats

TADMasterPlus accepts the two most prevalent Hi-C data formats: sparse matrices, where the format of the matrix is bin1, bin2, then interaction frequency, and the n-by-n matrix originally used by Lieberman *et al.* [1,8]. The cool format [2] which includes more data than on a traditional sparse matrix, such as edge weights. The hierarchal data format version 5 (.h5) [3] which stores a broad spectrum of data while being optimized for

storage. Finally, the .hic format [4] that is a compressed binary format allowing for storage of multiple contact matrices efficiently.

Normalization

Hi-C data that is submitted can be normalized to reduce noise and bias. The normalization is performed on sparse matrix that is either provided or extracted from the supplied input. For TAD Methods that require a non-sparse or n-by-n format the normalization is handled by the TAD method. A brief overview of each supported normalization is shown below:

Sequential component normalization (SCN) [5]

SCN is the method of going through the matrix and reading in values and determining probabilities. These probabilities are expressed as values between 0 and 1, being real non-negative numbers.

Iterative correction and eigenvector decomposition (ICE) [6]

ICE decomposes contact maps into a matrix of biases and contact probabilities. It also assumes all loci should have equal visibility, which improves the performance of modelling when applied across the matrix. From this a higher order organization can be developed.

Knight-Ruiz (KR) [7]

KR takes the non-negative input matrix and tries to find a proportion so the sums of all rows and columns are equal to 1. The values produced are chains of probabilities, or Markov Chain's, which are probabilities of an event based on prior events. With a double stochastic matrix, the chains are uniform.

Vanilla Coverage (VC) [8]

VC takes a value in a matrix and divided it by the sum of the column along with the sum of the row. It reduces noise by taking the matrix values and making them a product of the surrounding interaction frequencies, averaging them out.

Median contact frequency scaling (MCFS) [9]

MCFS takes values between two coordinates in the matrix. The median contact frequency is calculated for those two points, and the values in those coordinates are divided by the median contact frequency.

TAD Methods

Hi-C data can be analyzed by a multiple statistical, probabilistic, and clustering methods in order to identify TADs. A brief overview of each supported TAD method is shown below with the hyper parameters used. If a method only support specific Hi-C data formats and normalization methods, it is also noted:

Armatus [10]

TADs are acquired through dynamic programming identification of domains. These domains are across multiple resolutions, then derive to a consensus set of domains that span all resolutions. Armatus is run using an adjustable max gamma with the default set to 0.2. The step size is set to 0.025 and a minimum number of samples of 100 to compute the mean.

Arrowhead [11]

Domains are found using dynamic programming where the likelihood of a position being the corner of a domain is calculated. The peak values in the matrix are analyzed, with corresponding peaks being labelled as a domain. Arrowhead only works with .hic data format and can be normalized by either KR or VC. Arrowhead is run with the default parameters with the exception of ignoring sparce data inputs. The latter allows Arrowhead to compute results on a wider range of data inputs, however the resulting TADs may not be ideal.

CaTCH [12]

CaTCH stratifies a contact matrix into a series of smaller contact matrices by setting the insulation thresholds, or difference between interaction frequency thresholds. As the threshold amount decreases, borders between contacts regions decrease and thus a hierarchal structure of differentially insulated domains is created. CaTCH is run with the default parameters and searches for TADs within a reciprocal insulation range of 0.55 to 0.65.

ClusterTAD [13]

After normalizing the contact matrix, features for clustering are created from the length of the row and columns, however only one half of the matrix is considered due to it being symmetrical. Then a hierarchal cluster analysis is performed where TADs are derived from contact clusters. ClusterTAD is not run with any parameters, which results in

clustering and re-clustering to find TADs. The best TADs based on the quality score are presented for analysis.

Insulation Score [14]

Insulation score sums the contact frequencies around a particular locus using a rectangular sliding window. TADs are determined by a fixed contact count threshold, where regions exceeding threshold are classified by TADs. Three thresholds are used to identify TADs by insulation score. These thresholds are 40, 50, and 60 percent of the maximum found insulation score value. This approach is similar to the default approach of TADTool [15].

DI [16]

Directional index is a simple statistical method for deriving the upstream or downstream interaction bias. TADs can be identified by strongly directionally biased loci. Similar to Insulation Score the default approach proposed by TADTool[15] was utilized; a threshold determines what is statistically significant and were set similar to 40, 50, and 60 percent of the maximum found directional index value.

GMAP [17]

Gmap normalizes the interaction frequency, and a Gaussian mixture model is applied to the matrix to identify intra and inter chromatin domains. Choosing a bin size, the algorithm performs a proportion test between the inter-domain contact count and intra-domain contact count. Block boundaries are established to identify tads. By default, GMAP only utilizes resolution of the dataset to identify hierarchical domains

HiCExplorer [18]

HiCExplorer uses high resolution contact matrices to highlights and identifies important boundaries in contact matrices that can be used to identify TADs using machine learning. HiCExplorer only accepts cool, h5, or hic data formats. Further, it is only compatible with KR and ICE normalization provided by the tools hicCorrectMatrix function. HiCExplorer provides seven tunable parameters for TAD identification, however TADMaster utilizes the default parameters.

HiCseg [19]

HiCseg utilizes segmentation of a Hi-C contact map, the matrix is divided into smaller domains where associated interaction frequencies are grouped. HiCseg is executed using

a maximum number of change points set at 1000, the distribution of the data is set to a Gaussian distribution, and a block-diagonal is used for the model type.

IC-Finder [20]

IC-Finder Uses a hierarchal clustering method to analyze contact matrices for the locations of groups of interaction frequencies which are close together and share the same pattern. IC-Finder is executed using segmentation based on SigmaZero set to 5 to control the merging of two clusters.

SpectralTAD [21]

Using a sliding window-based approach, SpectralTAD performs a spectral clustering framework. TADs are identified using gaps between consecutive eigenvectors. SpectralTAD is executed using one level for TAD hierarch with the filtering based on the z-scores of eigenvector gaps.

TopDom [22]

A value is calculated based upon the average contact frequency, upstream and downstream, creating a curve that runs the length of the chromosome. It finds TAD boundaries from local minimums and filters false positives by using statistical analysis. TopDom is executed with and adjustable number of bins, and the default is set to 5.

Results

Running Time Estimates

The TADMaster web server runs on a HP G7-DL980, Intel(R) Xeon(R) CPU E7- 4870 @ 2.40GHz server with 120 Cores,1 Terabyte (TB) of RAM and with 40 TB of storage space.

TADMaster: Time from Job Submission to Visualization

The time it takes to upload a job with TADMaster is largely dependent on the size and format of the contact matrix. Jobs that do not include a contact matrix are typically completed within a couple minutes. If the job includes a large contact matrix file in a form that needs to be unpacked, such as the. mcool format, then the completion time can take between 10 to 30 minutes. The time it takes to visualize the results is also dependent on the number of TADs datasets and the size of the contact matrix. A sample of load times are provided in Supplementary Table S1 for human Embryonic Stem Cell (hESC) chromosomes contact matrices at 40KB resolution from Dixon et al. [16].

Table S1: Initial time to load visualize analysis results from a sample of chromosomes with between 12 to 14 TADs datasets displayed.

**Chromosome**	**Analysis Load Time (secs)**	**Matrix Size (KB)**
**1**	**55**	**74872**
**4**	**25**	**44908**
**10**	**25**	**22517**
**19**	**20**	**5000**

TADMaster Plus: Time from Job Submission to Visualization

The time it takes to upload a job with TADMaster Plus is largely dependent on the size and format of the contact matrix. Similar to TADMaster, the format of the contact matrix determines the time it takes to be unpacked for normalization. However, this is only a significant portion of run-time if few normalizations and TAD identification methods are selected. Further, the size and number of selected methods are the largest contributor to completion time. A sample of job runtimes with five normalization and ten TAD identification methods selected are provided in Table S2 for 40KB human Embryonic Stem Cell (hESC) [16]. On the table, methods not marked with an asterisk run in parallel; the time documented is approximately the time it takes to process the TAD method on five normalizations algorithms. The jobs marked with an asterisk are run serially on the server; the time documented is approximately the time it takes to process the TAD callers on a single normalization method. The jobs not run in parallel consume more computational resources, especially memory, and we found out that it is counterproductive to run these methods in parallel; hence, we execute them serially. Also, on the Table S2, we show the times it takes to normalize the input data uploaded for these datasets on all the five normalization algorithms that we provide.

Table S2: Comparison of time to run the Normalization and TADCaller algorithms between chromosome 8 (with size = 26,292 KB) and chromosome 19 (with size = 5,000

KB). The chromosomes were provided in square matrix formats, and both chromosomes were run with all normalization methods selected.

**Process**	**Chromosome 8 Running Time**	**Chromosome 19 Running Time**
**Five Normalization Algorithms**	1hr 38 min	7 min 8 sec
**Armatus**	1 min 20 sec	25 sec
**TopDom**	3 min 27 sec	51 sec
**GMAP**	2 min 11 sec	55 sec
**IC-Finder**	10 min 17 sec	2 min 5 sec
**HiCseg**	11 min 44 sec	5 min 12 sec
**Insulation Score**	2 min 5 sec	55 sec
**DI**	7 min 18 sec	1 min 30 sec
**Spectral**	1 min 20 sec	25 sec
**CaTCH***	4 mins 50 sec	2 min 19 sec
**ClusterTAD***	3 hrs 12 min	22min 11 sec

Discussion

TADMaster provides a platform for users to compare Bed files generated by TAD algorithms for a specific condition. In addition, one of the other strengths of TADMaster is to allow users to compare TADs generated from any source–and unique and common for these sources– to get insights into the relationships between the TADs from these cases. As an example, in this revised manuscript we compared TADs generated for chromosome 19 of human Embryonic Stem Cell (hESC) at 40KB resolution [16] for Spectral TAD for datasets generated across multiple normalizations, which are VC, ICE, MCFS, SCN, KR, and no normalization. The result of the analysis is available accessible at this link: http://biomlearn.uccs.edu/TADMaster/visualize_example/608/

With these results users can compare, for instance the impact of the normalization algorithm on the TADs detected by different algorithms. This can easily be transferred to studies interested in comparing a normal or cancerous cell dataset and how the TADs detected from both cases would differ.

REFERENCES

1. Van Berkum, N. L., Lieberman-Aiden, E., Williams, L., Imakaev, M., Gnirke, A., Mirny, L. A., Dekker, J. and Lander, E. S. (2010). Hi-C: a method to study the three-dimensional architecture of genomes. *JoVE (Journal of Visualized Experiments)*, (39), e1869.
2. Abdennur, N., and Mirny, L. A. (2020). Cooler: scalable storage for Hi-C data and other genomically labeled arrays. *Bioinformatics*, *36*(1), 311-316.
3. Mason, C. E., Zumbo, P., Sanders, S., Folk, M., Robinson, D., Aydt R, Gollery M., Welsh M., Olson N.E., and Smith, T. M. (2010). Standardizing the next generation of bioinformatics software development with BioHDF (HDF5). In *Advances in Computational Biology* (pp. 693-700). Springer, New York, NY.
4. Durand, N. C., Shamim, M. S., Machol, I., Rao, S. S., Huntley, M. H., Lander, E. S., and Aiden, E. L. (2016). Juicer provides a one-click system for analyzing loop-resolution Hi-C experiments. *Cell systems*, *3*(1), 95-98.
5. Cournac, A., Marie-Nelly, H., Marbouty, M., Koszul, R., and Mozziconacci, J. (2012). Normalization of a chromosomal contact map. *BMC genomics*, *13*(1), 1-13.
6. Imakaev, M., Fudenberg, G., McCord, R. P., Naumova, N., Goloborodko, A., Lajoie, B. R., Dekker J., and Mirny, L. A. (2012). Iterative correction of Hi-C data reveals hallmarks of chromosome organization. *Nature methods*, *9*(10), 999-1003.
7. Knight, P. A., and Ruiz, D. (2013). A fast algorithm for matrix balancing. *IMA Journal of Numerical Analysis*, *33*(3), 1029-1047.
8. Lieberman-Aiden, E., van Berkum, N.L., Williams, L., Imakaev, M., Ragoczy, T., Telling, A., Amit, I., Lajoie, B.R., Sabo, P.J., Dorschner, M.O. *et al.* (2009)

Comprehensive mapping of long-range interactions reveals folding principles of the human genome. *Science*, **326**, 289-293.

9. Kumar, R., Sobhy, H., Stenberg, P., and Lizana, L. (2017). Genome contact map explorer: a platform for the comparison, interactive visualization and analysis of genome contact maps. *Nucleic acids research*, *45*(17), e152-e152.
10. Filippova, D., Patro, R., Duggal, G., and Kingsford, C. (2014). Identification of alternative topological domains in chromatin. *Algorithms for Molecular Biology*, *9*(1), 1-11.
11. Rao, Suhas SP, Miriam H. Huntley, Neva C. Durand, Elena K. Stamenova, Ivan D. Bochkov, James T. Robinson, Adrian L. Sanborn A.L., Machol I., Omer A.D., Lander E.S., and Aiden E.L. (2014). A 3D map of the human genome at kilobase resolution reveals principles of chromatin looping. *Cell* 159, no. 7 (2014): 1665-1680.
12. Zhan, Y., Mariani, L., Barozzi, I., Schulz, E. G., Blüthgen, N., Stadler, M., Guido, T, and Giorgetti, L. (2017). Reciprocal insulation analysis of Hi-C data shows that TADs represent a functionally but not structurally privileged scale in the hierarchical folding of chromosomes. *Genome research*, *27*(3), 479-490.
13. Oluwadare, O., and Cheng, J. (2017). ClusterTAD: an unsupervised machine learning approach to detecting topologically associated domains of chromosomes from Hi-C data. *BMC bioinformatics*, *18*(1), 1-14.
14. Crane, E., Bian, Q., McCord, R. P., Lajoie, B. R., Wheeler, B. S., Ralston, E. J., Uzawa, S., Dekker, J., and Meyer, B. J. (2015). Condensin-driven remodelling of X chromosome topology during dosage compensation. *Nature*, *523*(7559), 240–244.
15. Kruse, K., Hug, C. B., Hernández-Rodríguez, B., and Vaquerizas, J. M. (2016). TADtool: visual parameter identification for TAD-calling algorithms. *Bioinformatics*, *32*(20), 3190-3192.
16. Dixon, J.R., Selvaraj, S., Yue, F., Kim, A., Li, Y., Shen, Y., Hu, M., Liu, J.S. and Ren, B. (2012) Topological domains in mammalian genomes identified by analysis of chromatin interactions. *Nature*, **485**, 376-380.
17. Yu, W., He, B., and Tan, K. (2017). Identifying topologically associating domains and subdomains by Gaussian mixture model and proportion test. *Nature communications*, *8*(1), 1-9.
18. Ramírez, F., Bhardwaj, V., Arrigoni, L., Lam, K. C., Grüning, B. A., Villaveces, J., and Habermann B, Akhtar A, and Manke, T. (2018). High-resolution TADs reveal DNA sequences underlying genome organization in flies. *Nature communications*, *9*(1), 1-15.
19. Lévy-Leduc, C., Delattre, M., Mary-Huard, T., and Robin, S. (2014). Two-dimensional segmentation for analyzing Hi-C data. *Bioinformatics*, *30*(17), i386-i392.
20. Haddad, N., Vaillant, C., and Jost, D. (2017). IC-Finder: inferring robustly the hierarchical organization of chromatin folding. *Nucleic acids research*, *45*(10), e81-e81.
21. Cresswell, K. G., Stansfield, J. C., and Dozmorov, M. G. (2020). SpectralTAD: an R package for defining a hierarchy of topologically associated domains using spectral clustering. *BMC bioinformatics*, *21*(1), 1-19.

22. Shin, H., Shi, Y., Dai, C., Tjong, H., Gong, K., Alber, F., and Zhou, X. J. (2016). TopDom: an efficient and deterministic method for identifying topological domains in genomes. *Nucleic acids research*, *44*(7), e70-e70.
