# Supplementary material for: TADMaster: a comprehensive web-based tool for the analysis of topologically associated domains
Source: BMC Bioinformatics. 2022 Nov 4;23:463. doi: 10.1186/s12859-022-05020-2 (PMC9636664; doi:10.1186/s12859-022-05020-2)
Supplement: Supplementary file 2 — Additional file 2: Table S1. Initial time to load visualize analysis results from a sample of chromosomes with between 12 to 14 TADs datasets displayed. [file 12859_2022_5020_MOESM2_ESM.docx]

**Table S1**: Initial time to load visualize analysis results from a sample of chromosomes with between 12 to 14 TADs datasets displayed.

| **Chromosome** | **Analysis Load Time (secs)** | **Matrix Size (KB)** |
| --- | --- | --- |
| **1** | **55** | **74872** |
| **4** | **25** | **44908** |
| **10** | **25** | **22517** |
| **19** | **20** | **5000** |
