# Supplementary material for: TADMaster: a comprehensive web-based tool for the analysis of topologically associated domains
Source: BMC Bioinformatics. 2022 Nov 4;23:463. doi: 10.1186/s12859-022-05020-2 (PMC9636664; doi:10.1186/s12859-022-05020-2)
Supplement: Supplementary file 3 — Additional file 3: Table S2.Comparison of time to run the Normalization and TADCaller algorithms between chromosome 8 (with size = 26,292 KB) and chromosome 19 (with size = 5000 KB). The chromosomes were provided in square matrix formats, and both chromosomes were run with all normalization methods selected. [file 12859_2022_5020_MOESM3_ESM.docx]

**Table S2**: Comparison of time to run the Normalization and TADCaller algorithms between chromosome 8 (with size = 26,292 KB) and chromosome 19 (with size = 5,000 KB). The chromosomes were provided in square matrix formats, and both chromosomes were run with all normalization methods selected.

| **Process** | **Chromosome 8 Running Time** | **Chromosome 19 Running Time** |
| --- | --- | --- |
| **Five Normalization Algorithms** | 1hr 38 min | 7 min 8 sec |
| **Armatus** | 1 min 20 sec | 25 sec |
| **TopDom** | 3 min 27 sec | 51 sec |
| **GMAP** | 2 min 11 sec | 55 sec |
| **IC-Finder** | 10 min 17 sec | 2 min 5 sec |
| **HiCseg** | 11 min 44 sec | 5 min 12 sec |
| **Insulation Score** | 2 min 5 sec | 55 sec |
| **DI** | 7 min 18 sec | 1 min 30 sec |
| **Spectral** | 1 min 20 sec | 25 sec |
| **CaTCH*** | 4 mins 50 sec | 2 min 19 sec |
| **ClusterTAD*** | 3 hrs 12 min | 22min 11 sec |
